# Comparison of outcomes in small bowel surgery for Crohn’s disease: a retrospective NSQIP review

**DOI:** 10.1007/s00384-024-04661-4

**Published:** 2024-07-29

**Authors:** Oguz AZ. Aras, Apar S. Patel, Emma K. Satchell, Nicholas J. Serniak, Raphael M. Byrne, Burt Cagir

**Affiliations:** 1 Department of Surgery, Guthrie Clinic, Guthrie Robert Packer Hospital, 1 Guthrie Square, Sayre, PA 18840 USA; 2 Department of Internal Medicine, TriStar Centennial Medical Center, Nashville, TN USA; 3 Department of Surgery, Geisinger Health System, Danville, PA USA

**Keywords:** Small bowel surgery, Crohn’s, 30-day outcomes, NSQIP review

## Abstract

**Introduction:**

Despite advances in medical therapy, approximately 33% of Crohn’s disease (CD) patients will need surgery within 5 years after initial diagnosis. Several surgical approaches to CD have been proposed including small bowel resection, strictureplasty, and combined surgery with resection plus strictureplasty. Here, we utilize the American College of Surgeons (ACS) national surgical quality registry (NSQIP) to perform a comprehensive analysis of 30-day outcomes between these three surgical approaches for CD.

**Methods:**

The authors queried the ACS-NSQIP database between 2015 and 2020 for all patients undergoing open or laparoscopic resection of small bowel or strictureplasty for CD using CPT and IC-CM 10. Outcomes of interest included length of stay, discharge disposition, wound complications, 30-day related readmission, and reoperation.

**Results:**

A total of 2578 patients were identified; 87% of patients underwent small bowel resection, 5% resection with strictureplasty, and 8% strictureplasty alone. Resection plus strictureplasty (combined surgery) was associated with the longest operative time (*p* = 0.002). Patients undergoing small bowel resection had the longest length of hospital stay (*p* = 0.030) and the highest incidence of superficial/deep wound infection (44%, *p* = 0.003) as well as the highest incidence of sepsis (3.5%, *p* = 0.03). Small bowel resection was found to be associated with higher odds of wound complication compared to combined surgery (OR 2.09, *p* = 0.024) and strictureplasty (1.9, *p* = 0.005).

**Conclusion:**

Our study shows that various surgical approaches for CD are associated with comparable outcomes in 30-day related reoperation and readmission, or disposition following surgery between all three surgical approaches. However, small bowel resection displayed higher odds of developing post-operative wound complications.

## Introduction

Crohn’s disease (CD) is a chronic inflammatory bowel disease that can affect any region of the gastrointestinal tract from the oropharynx to the anus. It is characterized by inflammatory skip lesions that can produce transmural inflammation along the digestive system. CD can be further categorized into three phenotypic presentations via imaging, endoscopy, and clinical presentation: inflammatory, penetrating, and fibro-stenotic. Secondary to CD phenotype, a number of GI pathologies can present in patients including intestinal thickening, abscess, fistula, stricture, bowel obstruction, and bowel perforation [1].

Generally, first-line treatment for CD is medical therapy. Medications are used to address the autoimmune nature of CD via chronic immunosuppression. Medications used include glucocorticoids, biologic monoclonal antibodies (anti-tumor necrosis factor (TNF), anti-integrin, or anti-interleukins), and immunomodulators (methotrexate, 6-mecaptopurine, azathioprine) [2]. Notably, monoclonal antibodies have become the foundation of treatment along with glucocorticoids. Patients with moderate-severe disease have shown response rates of 86% for biologics within 5–7 days of treatment after inadequate response to 5–7 days of glucocorticoids [3]. Medical therapy aims to decrease probability/severity of relapse in patients with quiescent disease and establish remission/decrease severity with patients with active disease. However, a drawback of biologic therapy is increased risk of infection due to immune suppression, with an odds ratio (OR) of 1.2 for any infection and OR of 1.9 for opportunistic infection vs. common comparator-placebo group [4].

Nevertheless, despite advances in medical therapy and treatment guidelines that aim to avoid surgery, 33% of Crohn’s patients will need surgery 5 years after initial diagnosis. Forty-seven percent will need surgery at 10 years [5].

*Inflammatory phenotype* of CD resulting in severe acute colitis (≥ 6 stools/day and systemic inflammatory response syndrome (SIRS)) not responding to medical therapy, and signs of bowel perforation, or impending perforation requires surgical intervention [6]. In these cases, bowel resection aims to remove diseased bowel and preserve normal bowel. There is no observed benefit when removing microscopically negative margins vs. limited  resections in CD [7].

*Penetrating phenotype* of CD can result in abscess formation, fistulas, and free perforation [8–10]. Abscess formation in setting of CD can be treated with a combination of antibiotics, percutaneous drainage, and bowel resection, depending on clinical presentation [9]. Fistulas are approached initially with medical (anti-TNF) therapy, resulting in 50% of fistula resolution, while 47% of patients required additional surgical resection within 5 years [11]. Penetrating phenotype with free perforation should be surgically treated with bowel resection [8].

*Fibro-stenotic phenotype* can result in fibrotic strictures anywhere along the intestinal tract and subsequently cause bowel obstruction. The initial approach for fibro-stenotic strictures is endoscopic dilation. Endoscopic dilation is utilized in the absence of fistula/penetrating disease and indicated for short segment (< 5 cm) strictures [12]. Success rates for endoscopic stricture dilation are nearly 90%; however, repeat endoscopic dilation is required in the majority of patients in the following 5 years [13]. For fibro-stenotic strictures that are not amenable to endoscopic dilation, surgical intervention is utilized. Resection or strictureplasty can be considered.

Compared to resection, strictureplasty is useful in preserving bowel length and maintaining absorptive ability, particularly in patients at risk for short-bowel syndrome. It is also favored in patients with multiple strictures separated by lengths normal of small bowel. Strictureplasty should be avoided in bowel regions of active inflammation, perforation, poor nutritional status, dysplasia, and malignancy [14, 15]. There are three common  approaches to strictureplasty: Heineke-Mikulicz, Finney, and isoperistaltic side-to-side strictureplasty used for strictures that are < 10 cm, 10–15 cm, and > 25 cm in length, respectively. After strictureplasty, 90% of CD recurrence occurs at sites distant to strictureplasty with a 3% site-specific recurrence. Several centers have even shown complete endoscopic disease remission at earlier strictureplasty sites [16].

Although strictureplasty involves notable advantages, it does have a relatively narrower “use-case” compared to resection in CD. It is utilized primarily for fibro-stenotic strictures not amenable to endoscopic dilation and without the contraindications outlined previously. Strictureplasty is not utilized to resolve inflammatory or penetrating presentations for CD [6].

Early post-operative complications arising from strictureplasty are similar to complications of bowel resection. Complications include wound infection, abscess, sepsis, anastomotic leak, perforation, enterocutaneous fistula, luminal bleeding, and early reoperation at an estimated combined rate of 13% [17–19]. Often strictureplasty and resection will be utilized in combination to address the presentation of CD in the operating room [19].

Although many studies have described ileocecectomy and colon resection, far fewer have evaluated surgical intervention on other regions of the small bowel, especially strictureplasty.

On this topic, there have been several institutional studies and systematic reviews and meta-analysis that have evaluated outcomes of strictureplasty in CD [5, 20–25]. However only one systematic review and meta-analysis included in-study comparisons to outcomes of resection [24]. Similarly, several NSQIP (National Surgery Quality Improvement) database centered studies of CD resection have focused on comparison of open vs. laparoscopic approach to small bowel resection and pre-operative factors that predict negative outcomes [18, 26–30]. However, these NSQIP studies did not include a comparison with strictureplasty in their analysis.

Here, we aim to perform a comprehensive analysis of 30-day outcomes of patients undergoing surgery for CD using the NSQIP database comparing small bowel resection (SBR), strictureplasty (SPX), or combined surgery (small bowel resection + strictureplasty, CSX).

## Methods

### Data source

The National Surgical Quality Improvement Program (NSQIP) database was used. NSQIP contains patients nationwide aggregate data without hospital, provider, or patient-specific identifiers [31]. NSQIP participant user files for the years 2015–2020 were queried. We excluded years prior to International Classification of Diseases 10 (ICD-10) code use due to the ability to precisely identify intestinal obstruction secondary to CD with ICD-10. NSQIP is a de-identified database, therefore Institutional Review Board (IRB) review was neither sought nor required.

### Patient population

Patients with a diagnosis code of K50.0 through K50.9 undergoing open small bowel resection (Current Procedural Terminology (CPT) code 44,120; 44,125; 44,130), minimally invasive small bowel resection (CPT code 44,202), or strictureplasty (CPT code 44,615) were selected. Patients were divided based on the operative approach: bowel resection only, strictureplasty only, and bowel resection with strictureplasty (combined surgery). Patients who underwent ileocecectomy were excluded. A total of 2578 patients were identified.

### Covariates

Demographic factors were compared between the three operative groups: age, sex, body mass index (BMI), and race. Patient’s past medical history included hypertension (HTN), smoking status, pre-operative use of corticosteroids, diabetes mellitus (DM), dyspnea, history of cancer, diagnosis of chronic obstructive pulmonary disease (COPD), ascites, functional status, weight loss (> 10% in 3 months preceding), bleeding disorder, ASA physical status class, and anemia. We also reported modified frailty index (mFI) for each patient. mFI is computed using five variables: functional status, diabetes, hypertension requiring medication, history of COPD, and history of CHF. The presence of these factors constitutes a score of 1, and all component scores are added together to calculate a total score. The mFI scores were grouped as 0, 1, or ≥ 2.

### Outcomes of interest

Perioperative and 30-day post-operative outcomes included surgery as elective or emergent, laparoscopic, operative time, hospital length of stay, wound classification, surgical site infection ([SSI] as superficial, deep, and organ/space), sepsis, septic shock, hospital transfer status, ventilator use, transfusion, discharge disposition, 30-day related readmission, and 30-day related reoperation.

### Statistical analyses

Categorical variables were compared among the groups using chi-square or Fischer’s exact test, while numeric variables were compared among the three groups using ANOVA (parametric) or Kruskal-Wallis (non-parametric) test. Analysis was performed to assess impact of surgical procedure among all demographic and clinical characteristics including age group, sex, DM, BMI, weight loss, steroid use, ASA class, elective or emergent status, operative time, laparoscopic vs. open procedure, and hospital length of stay.

## Results

### Demographic and comorbidity characteristics

A total of 2578 patients were identified: 2245 patients (87%) underwent SBR, 125 patients (5%) underwent CSX, and 208 (8%) patients underwent SPX. Patients who underwent all types of surgery more likely to be younger compared to the older cohort (46%, 42%, and 12% for age 18–40, 41–65, and 65+, respectively, Table 1). Patients undergoing SBR were more likely to have higher BMI compared to CSX and SPX groups (Table 1). African American patients were less likely to receive SPX compared to white patients (4.2% vs. 9%, Table 1). Patients undergoing CSX were less likely to have HTN (8%) compared to SBR (18.2%) and SPX groups (15.4%, Table 1). All groups had different rates of smoking: 20.3% for SBR, 6.4% for CSX, and 13.5% for SPX (Table 1). Patients undergoing CSX were the most likely to be on steroids (72%), followed by SPX (69.2%), and finally SBR (56.1%, Table 1). Lastly, patients undergoing SBR were more likely to have mFI of ≥ 2 (2.4%, *n* = 54/2245) compared to those undergoing SPX (0.5%, *n* = 1/208) and CSX (*n* = 0/125, Table 1).

**Table 1:** Demographic characteristics and comorbidities

		Resection only (*n* = 2245)	Resection with strictureplasty (*n* = 125)	Strictureplasty only (*n* = 208)	*p﻿*-value
Age	Mean (std)	44.8 (16.1)	41.0 (14.4)	43.1 (15.2)	**0.016**
	Range	18–90	18–78	18–82	
Age groups (%)	18–40	1009 (44.9)	71 (56.8)	108 (51.9)	**0.013**
	41–65	949 (42.3)	47 (37.6)	80 (38.5)	
	65+	287 (12.8)	7 (5.6)	20 (9.6)	
Sex (%)	Female	1050 (46.8)	52 (41.6)	98 (47.1)	0.522
	Male	1195 (53.2)	73 (58.4)	110 (52.9)	
BMI (%)	Mean (SD)	25.127 (6.1)	23.214 (4.7)	23.924 (5.6)	**< 0.001**
	Range	12.8–65.1	15.8–51.5	13.5–52.7	
	*n* missing	37	0	1	
Race (%)	American Indian or Alaska Native	4 (100)	0	0	**< 0.001**
	Asian	27 (84)	4 (13)	1 (3)	
	African American	202 (92)	8 (3.6)	9 (4.1)	
	Native Hawaiian or Pacific Islander	3 (100)	0	0	
	White	1681 (85.4)	107 (5.4)	179 (9.1)	
	Unknown	328 (93.2)	5 (1.4)	19 (5.4)	
Hypertension(%)	No	1837 (81.8)	115 (92.0)	176 (84.6)	**0.010**
	Yes	408 (18.2)	10 (8.0)	32 (15.4)	
Smoker (%)	No	1789 (79.7)	117 (93.6)	180 (86.5)	**< 0.001**
	Yes	456 (20.3)	8 (6.4)	28 (13.5)	
Steroid (%)	No	986 (43.9)	35 (28.0)	64 (30.8)	**< 0.001**
	Yes	1259 (56.1)	90 (72.0)	144 (69.2)	
mFI group (%)	0	1766 (79.0)	111 (88.8)	175 (84.1)	**0.012**
	1	416 (18.6)	14 (11.2)	32 (15.4)	
	≥ 2	54 (2.4)	0	1 (0.5)	

No significant associations were found for CD surgery SBR, SPX, CSX cohorts and a previous diagnosis of DM, dyspnea, previous disseminated cancer, COPD, ascites, weight loss, bleeding disorder, ASA class, anemia, and class of functional status (Table 4).

### Hospital and 30-day outcomes

Patients undergoing elective procedure were more likely to undergo CSX (80%) or SPX (79.8%), compared to SBR (65.6%, Table 2). Laparoscopic approach was used in 100% of patients undergoing SPX, 81.6% patients undergoing CSX and 72.2% of SBR (Table 2). Patients undergoing CSX had the longest operative time (209.6 min), followed by SPX (198.6 min), and SBR (182.5 min) (Table 2). Patients undergoing SBR had the longest length of stay (8.8 days), followed by CSX (7.8 days) and SPX (7.5 days, Table 3). Patients undergoing SBR had a higher rate of contaminated and dirty wounds (44%) compared to CSX (34%, Table 2). Patients undergoing SBR had a higher incidence of sepsis (3.5%) compared to SPX only (1.4%) and CSX (0%, Table 2).

**Table 2:** Summary of hospital stay and 30-day outcomes

		Resection only (*n* = 2245)	Resection with strictureplasty (*n* = 125)	Strictureplasty only (*n* = 208)	*p*-value
Elective surgery(%)	Unknown	3 (0.1)	0	1 (0.5)	**< 0.001**
	No	770 (34.3)	25 (20.0)	41 (19.7)	
	Yes	1472 (65.6)	100 (80.0)	166 (79.8)	
Laparoscopic procedure(%)	Yes	1621 (72.2)	102 (81.6)	208 (100)	**< 0.001**
	No	624(27.8)	23 (18.4)	0	
Operative time	Mean (std)	182.5 (101.4)	209.6 (95.4)	198.6 (93.0)	**0.002**
	Range	5–979	40–585	17–692	
Hospital length of stay	Unknown	26	1	3	**0.030**
	Mean (std)	8.8 (8.4)	7.8 (7.4)	7.5 (4.9)	
	Range	0–104	1–63	0–29	
Wound classification(%)	1-Clean	22 (1.0)	1 (0.8)	1 (0.5)	**0.003**
	2-Clean/contaminated	1234 (55.0)	81 (64.8)	125 (60.1)	
	3-Contaminated	541 (24.1)	28 (22.4)	62 (29.8)	
	4-Dirty/infected	448 (20.0)	15 (12.0)	20 (9.6)	
Sepsis(%)	No	2167 (96.5)	125 (100)	205 (98.6)	**0.033**
	Yes	78 (3.5)	0	3 (1.4)	

**Table 3:** Summary of hospital stay and 30-day outcomes of all three surgical approaches

**Covariate**	**Wound complication**	**30-day-related reoperation**	**30-day-related readmission**	**Prolonged length of stay**	**Routine discharge**
	OR [95% CI] (*p*-value)				
Group: small bowel resection only vs. combined procedure	2.09 [1.10–3.97] (**0.024**)	0.74 [0.27–2.02] (0.555)	1.54 [0.74–3.18] (0.245)	1.47 [0.92–2.36] (0.108)	0.36 [0.05–2.80] (0.328)
Group: strictureplasty only vs. combined procedure	1.02 [0.46–2.22] (0.970)	0.98 [0.28–3.42] (0.978)	1.15 [0.48–2.77] (0.748)	1.21 [0.69–2.12] (0.512)	0.50 [0.05–5.21] (0.565)
Group: strictureplasty only vs. small bowel resection only	0.52 [0.32–0.81] (**0.005**)	0.92 [0.49–1.7] (0.786)	0.71 [0.44–1.14] (0.157)	0.60 [0.44–0.80] (**0.031**)	1.3 [0.58–3.1] (0.490)
Emergency vs. elective	1.24 [0.84–1.83] (0.284)	0.95 [0.49–1.86] (0.881)	1.23 [0.77–1.98] (0.385)	2.63 [1.91–3.64] (**< 0.001**)	0.84 [0.40–1.77] (0.652)
Laparoscopic procedure	0.42 [0.30–0.59] (**< 0.001**)	1.38 [0.80–2.39] (0.251)	0.90 [0.64–1.26] (0.533)	0.55 [0.43–0.71] (< **0.001**)	1.87 [0.82–4.29] (0.139)
Operative time	1.002 [1.001–1.003] (**< 0.001**)	1.00 [1.00–1.00] (0.11)	1.002 [1.001–1.003] (**0.019**)	1.002 [1.001–1.003] (**< 0.001**)	1.00 [1.00–1.00] (0.173)
Any wound complication		16.98 [10.81–26.67] (**< 0.001**)	6.80 [5.08–9.10] (**< 0.001**)	2.85 [2.25–3.61] (**< 0.001**)	0.55 [0.32–0.96] (**0.035**)
Prolonged length of stay	1.07 [1.06–1.09] (**< 0.001**)	1.07 [1.05–1.09] (**< 0.001**)	0.97 [0.96–0.99] (**0.004**)		0.93 [0.92–0.95] (**< 0.001**)
Age groups = 41–65 vs. 18–40	0.89 [0.69–1.15] (0.369)	1.24 [0.79–1.94] (0.345)	1.10 [0.82–1.46] (0.537)	0.94 [0.76–1.16] (0.587)	0.42 [0.21–0.87] (**< 0.001**)
Age groups = 65+ vs. 18–40	0.82 [0.56–1.21] (0.323)	0.82 [0.40–1.68] (0.590)	0.93 [0.59–1.45] (0.741)	1.12 [0.83–1.52] (0.464)	0.11 [0.05–0.23] (**0.019**)
Male vs. female	0.89 [0.71–1.12] (0.310)	1.19 [0.79–1.78] (0.415)	0.79 [0.60–1.02] (0.072)	1.01 [0.84–1.22] (0.922)	1.35 [0.81–2.23] (0.246)
Diabetes	0.83 [0.44–1.56] (0.558)	1.13 [0.36–3.51] (0.836)	1.20 [0.62–2.30] (0.584)	0.86 [0.52–1.42] (0.547)	0.81 [0.32–2.03] (0.651)
BMI	1.01 [0.99–1.03] (0.278)	1.00 [0.97–1.04] (0.823)	1.01 [0.99–1.04] (0.180)	0.98 [0.97–1.00] (**0.045**)	0.97 [0.93–1.01] (0.153)
Smoker	1.06 [0.80–1.41] (0.684)	1.13 [0.69–1.85] (0.640)	1.04 [0.74–1.44] (0.838)	1.22 [0.97–1.54] (0.095)	1.41 [0.72–2.75] (0.312)
Weight loss	1.31 [0.93–1.85] (0.127)	0.37 [0.18–0.77] (**0.008**)	0.99 [0.64–1.52] (0.952)	2.14 [1.61–2.85] **(< 0.001**)	0.47 [0.24–0.92] (**0.028**)
Steroid use	1.18 [0.93–1.49] (0.176)	1.30 [0.85–1.98] (0.226)	1.24 [0.95–1.63] (0.119)	1.02 [0.84–1.24] (0.845)	1.21 [0.73–2.00] (0.456)
High ASA	1.07 [0.83–1.36] (0.607)	0.76 [0.49–1.19] (0.231)	1.19 [0.90–1.58] (0.230)	1.80 [1.48–2.20] (**< 0.001**)	0.64 [0.35–1.18] (0.155)

No significant associations were found for CD surgery SBR, SPX, CSX cohorts and transfer status, ventilator use, transfusion, superficial SSI, deep SSI, organ/space SSI, septic shock, discharge disposition, 30-day related readmission, and 30-day related reoperation (Table 5).

### Covariate analysis

#### Incidence of any wound complication

We grouped all wound complications including superficial, deep, and organ/space SSI under “any wound complication.” SBR compared to CSX (OR 2.09) and SPX (OR 1.9) was associated with increased odds of wound complication (Table 3). Other factors associated with higher odds of wound complication included longer hospital stay (OR 1.07) and longer procedure time, although narrowly (OR 1.002). Additionally, laparoscopic procedures had lower odds of wound complication (OR 0.42, Table 3).

#### 30-Day related reoperation

We did not find an association between procedure type (SBR, CSX, SPX) and incidence of 30-day related reoperation. A minor factor associated with a higher rate of 30-day related reoperation was longer length of stay (OR 1.07). A major factor was incidence of a wound complication (OR 16.98). Weight loss was negatively associated with 30-day related operation rate (0.37, Table 3).

#### 30-Day related readmission

We did not find an association between procedure type (SBR, CSX, SPX) and incidence of 30-day related readmission. Minor factors associated with 30-day related reoperation included longer operative time (OR 1.002) and shorter hospital stay (OR 0.97). A major factor in 30-day readmission was incidence of a wound complication (OR 6.8, Table 3).

#### Length of stay

For the purpose of this analysis, “prolonged length of stay” was defined as length of stay longer than 75th percentile for all CD surgical cohorts: 9.5 days. SPX compared to SBR had lower odds of prolonged hospital stay (OR 0.60). Factors associated with prolonged length of stay included emergency admission (OR 2.63), wound complications (OR 2.85), presence of weight loss (OR 2.14), higher ASA category (OR 1.8), and longer operative time (OR 1.002). Laparoscopic procedures and a BMI class (≥ 25 kg/m^2^) were associated with lower odds of prolonged length of stay (OR 0.55 and OR 0.98, respectively, Table 3).

#### Disposition following surgery

For the purpose of this analysis “home routine” and “facility which was home” were grouped together under “routine.” All other categories were grouped under “non-routine.” We did not find any association between procedure type (SBR, CSX, SPX) and discharge disposition. Factors found to be associated with lower odds of routine discharge included older age (OR 0.42 for age 41–65 vs. 18–40, OR 0.11 for age 65 + vs. 18–40), weight loss (OR 0.47), longer length of stay (OR 0.93), and incidence of a wound complication (OR 0.55, Table 3).

## Discussion

In our study, we sought to compare the 30-day outcomes of patients undergoing surgery for CD including small bowel resection (SBR), combined surgery (CSX, small bowel resection + strictureplasty), and strictureplasty (SPX) using the NSQIP database. The following outcomes of interest were evaluated for all three surgical techniques: 30-day related reoperation or readmission, routine discharge, wound complication, and prolonged length of stay.

Measured outcomes for all three surgical techniques were comparable for 30-day related reoperation and readmission. Likewise, all three surgical approaches showed similar outcomes for patient disposition on hospital discharge.

Patients undergoing SBR for CD showed greater odds of any wound complication and sepsis versus CSX and SPX. However, we also found pre-operative clinical characteristics in SBR cohort that are typically associated with worse surgical outcomes. These factors include a significantly higher rate of smoking, HTN, mFI score, surgical wound classification, and a significantly lower rate of elective procedure, laparoscopic approach, and pre-operative glucocorticoid use.

The deleterious effect of smoking on CD has been well established. Seksik et al. [32] showed smoking increases time with active CD from for non-smokers (33%) compared to active smokers (41%). A 10-year follow-up in CD patients found that smokers had a threefold increased risk of surgery compared to non-smokers [33]. The effect of smoking status on surgical outcomes of CD surgery is however less clear.

A previous NSQIP study evaluating the effect of smoking status in SBR for CD showed a positive association between smoking and increased morbidity via infectious and pulmonary complications (OR 1.3 and 1.87, respectively) [34]. A strength of our analysis is the inclusion of CSX and SPX in addition to SBR surgery in Crohn’s patients. Our NSQIP analysis however does not demonstrate a significant relationship between smoking status and negative surgical outcomes for any of the three surgical techniques. This discrepancy suggests smoking status alone is not significantly contributory to negative surgical outcomes in CD. However, when combined with other comorbidities such as ≥ 2 mFI, HTN, and lower rates of pre-operative steroids and elective and laparoscopic surgery, it increases risk of wound complications and prolongs hospital stay.

A higher mFI score in the SBR cohort demonstrates higher pre-operative morbidity. A 2018 systematic review and meta-analysis of 16 studies across surgical disciplines with 683,487 patients found frail patients were more likely to experience wound complications (relative risk, RR 1.52), readmission (RR 1.61), discharge to skilled care (RR 2.15), and mortality (RR 4.19) [35]. Our study confirms two of these findings in relation to wound complications and prolonged hospital stay in association to increased frailty, but we do not observe increased rates of discharge to skilled care, 30-day related reoperation or readmission in CD patients undergoing surgery.

Odds of adverse outcomes in emergency surgery have been well established in the literature, generally an increase in the odds of any complication [36]. In our analysis, the association of emergency vs. elective procedure in all three types of CD surgery only show a significant association with prolonged length of stay. This is in contrast to emergency surgery being associated with a 40% increase in overall complications versus elective surgery for CD bowel resection in a recent systematic review and meta-analysis [37].

Glucocorticoids are some of the most commonly used medications in patients with high disease activity and severe colitis in CD [6]. Nonetheless, pre-operative use of glucocorticoids is a known risk factor for negative outcomes after bowel surgery in a dose-dependent manner and therefore pre-operative optimization involves weaning of steroids [38]. In our analysis, CSX and SPX cohorts have a similar rate of elective surgery of approximately 80% compared to 65% in SBR. We propose a lower rate of pre-operative steroid use is secondary to lower rate of elective surgery in SBR cohort. This is likely due to the high probability of surgical intervention on admission (Tables 4 and 5).

**Table 4:** Pertinent negatives for demographic characteristics and comorbidities

		Resection only (*n* = 2245)	Resection with strictureplasty (*n* = 125)	Strictureplasty only (*n* = 208)	*p*-value
Diabetes(%)	Insulin	46 (2.0)	1 (0.8)	2 (1.0)	0.306
	Non-insulin	51 (2.3)	1 (0.8)	2 (1.0)	
	No diabetes	2148 (95.7)	123 (98.4)	204 (98.1)	
Dyspnea(%)	At rest	5 (0.2)	0 (0)	0 (0)	0.397
	Moderate exertion	56 (2.5)	1 (0.8)	2 (1.0)	
	No	2184 (97.3)	124 (99.2)	206 (99.0)	
Disseminated cancer(%)	No	2239 (99.7)	125 (100)	208 (100)	0.640
	Yes	6 (0.3)	0 (0)	0 (0)	
COPD(%)	No	2201 (98.0)	124 (99.2)	208 (100)	0.084
	Yes	44 (2.0)	1 (0.8)	0 (0)	
Ascites(%)	No	2240 (99.8)	124 (99.2)	207 (99.5)	0.402
	Yes	5 (0.2)	1 (0.8)	1 (0.5)	
Functional status(%)	Independent	2208 (98.4)	123 (98.4)	208 (100)	0.579
	Partially dependent	23 (1.0)	2 (1.6)	0 (0)	
	Totally dependent	5 (0.2)	0 (0)	0 (0)	
	Unknown	9 (0.4)	0 (0)	0 (0)	
Weight loss(%)	No	1998 (89.0)	111 (88.8)	188 (90.4)	0.823
	Yes	247 (11.0)	14 (11.2)	20 (9.6)	
Bleeding disorder(%)	No	2190 (97.6)	125 (100)	202 (97.1)	0.188
	Yes	55 (2.4)	0 (0)	6 (2.9)	
ASA class(%)	1-No disturb	25 (1.1)	1 (0.8)	3 (1.4)	0.222
	2-Mildly disturb	1157 (51.5)	72 (57.6)	110 (52.9)	
	3-Severe disturb	992 (44.2)	52 (41.6)	92 (44.2)	
	4-Life threat	65 (2.9)	0 (0)	2 (1.0)	
	5-Moribund	5 (0.2)	0 (0)	0 (0)	
	None assigned	1 (0)	0 (0)	1 (0.5)	
Anemia(%)	No anemia	1207 (53.8)	71 (56.8)	105 (50.5)	0.668
	Mild anemia	703 (31.3)	35 (28.0)	74 (35.6)	
	Moderate/severe anemia	335 (14.9)	19 (15.2)	29 (13.9)

**Table 5:** Pertinent negatives for hospital stay and 30-day outcomes

		Resection only (*n* = 2245)	Resection with strictureplasty (*n* = 125)	Strictureplasty only (*n* = 208)	*p*-value
Transfer status(%)	From acute care hospital inpatient	86 (3.8)	2 (1.6)	9 (4.3)	0.576
	Admitted from home	2095 (93.3)	121 (96.8)	198 (95.2)	
	Nursing home, intermediate, or chronic care	7 (0.3)	1 (0.8)	0 (0)	
	Outside emergency department	45 (2.0)	1 (0.8)	1 (0.5)	
	Transfer from other	10 (0.4)	0 (0)	0 (0)	
Ventilator(%)	No	2238 (99.7)	125 (100)	208 (100)	0.594
	Yes	7 (0.3)	0 (0)	0 (0)	
Transfusion(%)	No	2217 (98.8)	125 (100)	206 (99.0)	0.431
	Yes	28 (1.2)	0 (0)	2 (1.0)	
Superficial SSI(%)	No complication	2153 (95.9)	122 (97.6)	204 (98.1)	0.204
	Superficial SSI	92 (4.1)	3 (2.4)	4 (1.9)	
Deep SSI(%)	No	2143 (95.5)	123 (98.4)	204 (98.1)	0.066
	Yes	102 (4.5)	2 (1.6)	4 (1.9)	
Organ/space SSI(%)	No	2015 (89.8)	117 (93.6)	195 (93.8)	0.077
	Yes	230 (10.2)	8 (6.4)	13 (6.2)	
Septic shock(%)	No	2222 (99.0)	125 (100)	208 (100)	0.179
	Yes	23 (1.0)	0 (0)	0 (0)	
Discharge destination(%)	n-miss	20	1	2	0.961
	Against medical advice	3 (0.1)	0 (0)	0 (0)	
	Expired	10 (0.4)	1 (0.8)	0 (0)	
	Facility which was home	11 (0.5)	1 (0.8)	1 (0.5)	
	Home	2128 (95.6)	121 (97.6)	202 (98.1)	
	Hospice	1 (0)	0 (0)	0 (0)	
	Rehab	22 (1.0)	0 (0)	2 (1.0)	
	Separate acute care	10 (0.4)	0 (0)	0 (0)	
	Skilled care, not home	39 (1.8)	1 (0.8)	1 (0.5)	
30-day related readmission(%)	No	1952 (86.9)	116 (92.8)	188 (90.4)	0.067
	Yes	293 (13.1)	9 (7.2)	20 (9.6)	
30-day related reoperation(%)	No	2116 (94.3)	119 (95.2)	197 (94.7)	0.879
	Yes	129 (5.7)	6 (4.8)	11 (5.3)	

Hence, the SBR cohort differed along several confounding comorbidity characteristics compared to CSX and SPX. We therefore elaborate that the listed confounding variables, and not SBR surgical approach, predisposed these patients to higher rates of wound complication, prolonged length of stay, and higher rates of sepsis. Demographic characteristics and comorbidities, hospital course, and 30-day outcomes differ significantly between all three surgical approaches to CD are summarized in Table 6.

**Table 6:** Demographic characteristics and comorbidities, hospital course, and 30-day outcomes differ significantly between all three surgical approaches to CD

**Resection only**	**Resection with strictureplasty**	**Strictureplasty only**
↑ mean mFI ↑ rate of smoking ↑ rate of HTN ↑ mean BMI ↓ rate of pre-op steroid use	↓ mean mFI ↓ rate of smoking ↓ rate of HTN ↓ mean BMI ↑ rate of pre-op steroid use	↓ mean mFI → rate of smoking ↑ rate of HTN ↓ mean BMI ↑ rate of pre-op steroid use
↓ rate of elective procedure ↓ rate of laparoscopic procedure ↓ average operative time ↑ mean length of stay ↑ rate of contaminated and dirty wounds ↑ rate of sepsis	↑ rate of elective procedure → rate of laparoscopic procedure ↑ average operative time → mean length of stay ↓ rate of contaminated and dirty wounds ↓ rate of sepsis	↑ rate of elective procedure ↑ rate of laparoscopic procedure → average operative time ↓ mean length of stay ↓ rate of contaminated and dirty wounds ↓ rate of sepsis
↑ odds of wound complication		↓ odds of prolonged hospital stay ↓ rate of strictureplasty in African Americans

↑ significantly increased vs. other cohort(s), ↓ significantly decreased vs. other cohort(s), → significantly different versus other cohorts (significantly higher vs. ↓, but significantly lower vs. ↑)

We also found that African American patients undergo SPX at half the rate of White patients. This may be reflective of a racial disparity. Previous studies have demonstrated disparities in terms of both access to less invasive surgical options as well as in terms of outcomes for African American patients for common gastrointestinal surgical disease. Wood et al. [39] queried the NSQIP 2016 Participant Use Data File for all general surgical procedures and found African Americans patients were 11% less likely to undergo a laparoscopic procedure compared to White patients. An ACS-NSQIP analysis evaluating short-term outcomes after SBR, SPX, colorectal resection, colostomy, ostomy, and internal fistula closure for CD found significantly higher rate of complications for African American patients compared to non-African Americans (19% vs. 23.5%, respectively). This effect remained significant even after adjusting for pre-operative disease severity, smoking status, and BMI. Only after further adjusting for comorbid disease and ASA class did the significance of race lose statistical significance [40]. Our findings reflecting a lower utilization rate of SPX for African American patients compliments these findings and highlights the importance of increased vigilance in providing access to appropriate medical management and treatment of comorbid conditions in furthering the health of all patients with CD.

## Strengths and limitations

Our study has multiple strengths. This study is the only analysis to compare outcomes of small bowel resection (SBR) to strictureplasty (SPX) and strictureplasty with small bowel resection (CSX) using a NSQIP registry in the last 10 years [18]. The cross-national sample of collected patient data from a range of hospitals participating in the NSQIP database makes our results applicable to the US population. This can counterbalance a positive publication bias in previous primary research and its secondary inclusion in systematic reviews and meta-analyses.

However, this study also has several limitations. Our cohorts are defined using CPT codes; previous research has shown that coding errors can be common and can therefore lead to unreliable primary data [41]. We were unable to assess the impact of several pre-operative factors such as medical therapy for prior CD care and nutritional status (albumin). The analysis is limited to a 30-day post-operative period. Therefore, our study will lack further pertinent information regarding subsequent surgeries, complications, and related hospitalization after this 30-day period. There are also limitations to using the NSQIP database for comparing surgical outcomes for different racial cohorts. The NSQIP database does not capture information on socioeconomic status, location, type of medical insurance, and other health-seeking behaviors. [42].

A major limitation of this study is comparing surgeries for which there are differing indications. SBR in CD is indicated for several scenarios: exacerbation of inflammatory, penetrating, and fibro-stenotic phenotype and free perforation. This is compared to SPX indicated for fibro-stenotic phenotype excluding active inflammation, dysplasia, malignancy, and poor nutritional status as per surgical guidelines of the American Society of Colon and Rectal Surgeons [6].

In conclusion, our analysis showed significant differences in odds of developing post-operative wound complications and prolonged hospitalization for patients undergoing SBR compared to CSX and SPX for CD. We did not observe differences in 30-day related reoperation and readmission, or disposition following surgery between all three surgical approaches.

Of note, post-operative wound complications resulted in a 17-fold increase in odds of 30-day reoperation, a 6.8-fold increase in 30-day readmission, a 2.85-fold increase in prolonged hospitalization, and roughly half of patients not discharged to home, independent of surgical technique used. Laparoscopic procedures resulted in approximately 60% decrease in odds of wound complications and 45% decrease in prolonged hospitalization, independent of surgical technique used. Lastly, age group was a significant factor associated with 60% and 90% reduction in routine discharge in age groups 41–65 and 65+, respectively, when compared to patients aged 18–40. Age group was not associated with any other negative outcome evaluated.

## Data Availability

The American College of Surgeons, National Surgery Quality Improvement database is de-identified. Therefore, Institutional Review Board (IRB) review was neither sought nor required. The American College of Surgeons National Surgical Quality Improvement Program (ACS-NSQIP) and the hospitals participating in the ACS-NSQIP are the sources of the data used herein; they have not verified and are not responsible for the statistical validity of the data analysis or the conclusions derived by the authors.
